# OptimalTTF-1: Enhancing tumor treating fields therapy with skull remodeling surgery. A clinical phase I trial in adult recurrent glioblastoma

**DOI:** 10.1093/noajnl/vdaa121

**Published:** 2020-09-15

**Authors:** Anders Rosendal Korshoej, Slavka Lukacova, Yasmin Lassen-Ramshad, Christian Rahbek, Kåre Eg Severinsen, Trine Lignell Guldberg, Nikola Mikic, Mette Haldrup Jensen, Søren Ole Stigaard Cortnum, Gorm von Oettingen, Jens Christian Hedemann Sørensen

**Affiliations:** 1 Department of Neurosurgery, Aarhus University Hospital, Aarhus, Denmark; 2 Department of Clinical Medicine, Aarhus University, Aarhus, Denmark; 3 Department of Oncology, Aarhus University Hospital, Aarhus, Denmark; 4 Danish Centre for Particle Therapy, Aarhus University Hospital, Aarhus, Denmark; 5 Department of Neuroradiology, Aarhus University Hospital, Aarhus Denmark; 6 Department of Neurology, Aarhus University Hospital, Aarhus, Denmark; 7 Department of Oncology, Aalborg University Hospital, Aalborg, Denmark

**Keywords:** craniectomy, glioblastoma, neuro-oncology, neurosurgery, tumor treating fields

## Abstract

**Background:**

Preclinical studies suggest that skull remodeling surgery (SR-surgery) increases the dose of tumor treating fields (TTFields) in glioblastoma (GBM) and prevents wasteful current shunting through the skin. SR-surgery introduces minor skull defects to focus the cancer-inhibiting currents toward the tumor and increase the treatment dose. This study aimed to test the safety and feasibility of this concept in a phase I setting.

**Methods:**

Fifteen adult patients with the first recurrence of GBM were treated with personalized SR-surgery, TTFields, and physician’s choice oncological therapy. The primary endpoint was toxicity and secondary endpoints included standard efficacy outcomes.

**Results:**

SR-surgery resulted in a mean skull defect area of 10.6 cm^2^ producing a median TTFields enhancement of 32% (range 25–59%). The median TTFields treatment duration was 6.8 months and the median compliance rate 90%. Patients received either bevacizumab, bevacizumab/irinotecan, or temozolomide rechallenge. We observed 71 adverse events (AEs) of grades 1 (52%), 2 (35%), and 3 (13%). There were no grade 4 or 5 AEs or intervention-related serious AEs. Six patients experienced minor TTFields-induced skin rash. The median progression-free survival (PFS) was 4.6 months and the PFS rate at 6 months was 36%. The median overall survival (OS) was 15.5 months and the OS rate at 12 months was 55%.

**Conclusions:**

TTFields therapy combined with SR-surgery and medical oncological treatment is safe and nontoxic and holds the potential to improve the outcome for GBM patients through focal dose enhancement in the tumor.

**Key Points**

- Craniectomy and small burr holes in the skull focally enhance the dose of TTFields.
- Craniectomy is safe in combination with TTFields and not a contraindication.
- TTFields in combination with craniectomy or small burr holes is safe and likely effective.

**Importance of the Study**

We present phase I data for a new, rational, and innovative intervention combining tumor treating fields (TTFields) with targeted skull remodeling surgery to enhance the field dose and clinical efficacy against recurrent glioblastoma. The concept builds on preclinical dosimetry studies and recent clinical evidence that the field dose correlates with overall survival. Our study is the first to successfully achieve a significant and personalized TTFields dose accumulation focally in the tumor and we have used dosimetry methods to illustrate the concept and its impact. The treatment is easy to understand and implement and potentially extends to all intracranial applications of TTFields. Our data suggest that the treatment is safe and effective (median overall survival 15.5 months and progression-free survival rate at 6 months is 36%). Based on these results, we have initiated a subsequent prospective randomized phase 2 clinical trial scheduled to begin recruitment in summer 2020 (NCT04223999).

Glioblastoma (GBM) is the most prevalent and severe primary adult brain cancer. Despite maximum safe resection, radiotherapy, and chemotherapy, the prognosis is dismal with a median overall survival (OS) of approximately 1 year for newly diagnosed cases.^1–4^ Recently, tumor treating fields (TTFields, Optune) were included as a Category 1 recommendation for patients with newly diagnosed GBM by the National Comprehensive Cancer Network in the United States.^5^ TTFields are low-intensity (1–3 V/m) and intermediate frequency (200 kHz) alternating fields that disrupt mitosis and inhibit tumor growth.^6^ A recent randomized controlled phase 3 trial (EF-14) established that TTFields result in a sustained OS benefit (OS = 20.9 vs 16.0 months, *P* < .001) and prolonged progression-free survival (PFS = 6.7 vs 4.0 months, *P* < .001) for newly diagnosed GBM when added to temozolomide maintenance therapy.^7^ Although efficacy benefits have been less profound for recurrent GBM (rGBM), a randomized controlled phase 3 trial (EF-11) has demonstrated superior toxicity for TTFields monotherapy compared to best practice medical oncological therapy alone.^8^ Subsequent retrospective studies and a meta-analysis have since indicated a significant survival benefit for both recurrent and newly diagnosed GBM.^9,10^

## TTFields Dosimetry and Dose Enhancement With Skull Remodeling Surgery

Different methods have been implemented to quantify the dose distribution of TTFields in the brain and provide strategies for treatment planning and response prediction. Although the efficacy of TTFields is influenced by multiple factors, such as frequency and spatial correlation of the fields,^6,11,12^ the field intensity distribution is commonly accepted as a surrogate measure of treatment dose. The field intensity correlates positively with the tumor kill rate in vitro^6^ and the OS, that is, high field intensities in the tumor lead to longer OS.^13^ The field distribution can be estimated using finite element methods^14–17^ based on personalized computational models constructed from MRI data, see Refs ^18^ and ^17^ for further details on TTFields dosimetry and its clinical implications.

**Figure 1.**
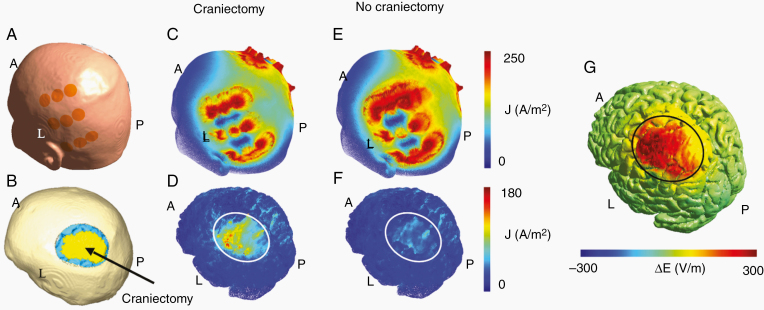
Distributions of current density and field intensity before and after SR-surgery. Panel A shows the position of one array pair on the surface of the patients head, while the craniectomy and underlying regions of interest (tumor, yellow; peritumoral border zone, blue) are shown in panel B. Craniectomy reduced the amount of current shunted through the skin between the arrays (panel C vs E) and redirects the current through the hole in the skull (panel D vs F) and toward the tumor. Panel G shows the change in field intensity induced on the surface of the brain. Craniectomy enhances the field intensity by approximately 300 V/m in the region of interest, while the dose in the surrounding brain tissue remains unaffected.

Using dosimetry methods, we previously showed that skull remodeling surgery (SR-surgery), including burr holes and minor craniectomies placed above the tumor region, provide a substantial (~70%) and highly focused enhancement of TTFields in the tumor without affecting the dose in the healthy tissues (Figure 1G).^14^ The skull defects serve as low-resistance pathways that facilitate current flow into the tumor region (Figure 1D) and prevent wasteful shunting of currents through the skin between the arrays, caused by shielding effects of the high-impedance skull (Figure 1C and E).

Here, we present the results of a proof-of-concept phase I clinical trial testing SR-surgery as a rational, innovative, and dose-enhancing method to improve TTFields therapy against rGBM.

## Methods

The trial was a prospective, open-label, single-center phase I trial investigating safety, feasibility, and preliminary efficacy of SR-surgery in combination with TTFields and best choice chemotherapy for rGBM (clinicaltrials.gov id NCT02893137). The study was performed at Aarhus University Hospital, Denmark, in the period October 1, 2016 to May 31, 2019. All study procedures were in accordance with the ethical standards of the Helsinki Declaration of the World Medical Association^19^ and followed guidelines for Good Clinical Practice (ICH-GCP), ISO-14155 standards, and relevant Danish regulations, see Supplementary Material S1 for further protocol details.

### Eligibility and Enrollment

Inclusion criteria were (1) age ≥18 years, (2) histopathological primary diagnosis of GBM using the WHO 2016 classification,^20^ (3) estimated survival ≥3 months, (4) supratentorial tumor location, (5) not a candidate for further radiotherapy, (6) first disease progression according to RANO criteria^21^ based on MRI performed no later than 4 weeks prior to enrollment, (7) Karnofsky performance score (KPS) ≥70, (8) ability to comply with TTFields, (9) significant expected benefit from feasible SR-surgery combined with TTFields, that is, (a) focal disease and (b) at least some part of the tumor or resection cavity had to be closer than 2 cm from the brain surface, and (10) signed written consent form.

Exclusion criteria were (1) pregnancy or nursing, (2) less than 4 weeks since radiation therapy, (3) infratentorial tumor, (4) implanted pacemaker, (5) programmable shunts, (6) deep brain stimulator, (7) refractory symptomatic epilepsy, (8) contraindications for SR-surgery, for example, bleeding diathesis or severe infection, (9) significant comorbidities, that is, significant liver function impairment, significant renal impairment, coagulopathy, thrombocytopenia, neutropenia, anemia, and (10) active participation in another therapeutic interventional clinical trial.

Patients were also excluded if rGBM could not be confirmed on histopathological examination of resected tissue after inclusion.

### Treatment Plan

All patients were treated with SR-surgery, TTFields, and physician’s choice medical oncological therapy.

#### SR-surgery and resection

**Figure 2.**
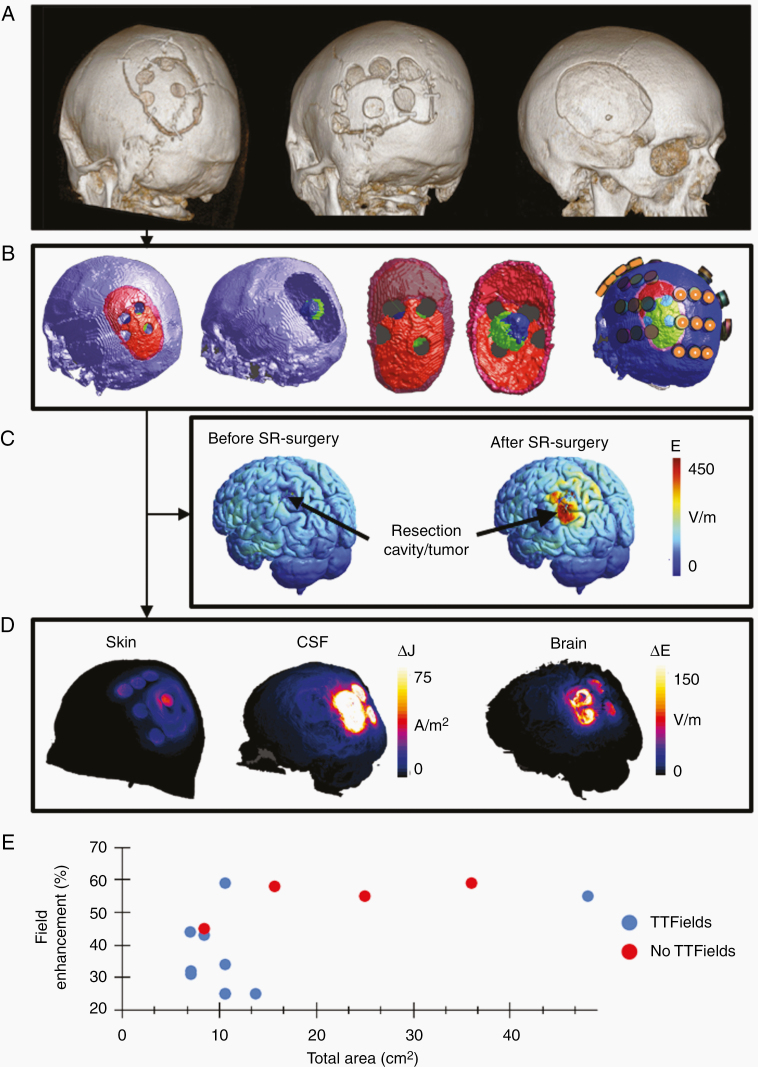
Examples of SR-surgery. Panel A shows 3 examples of SR-surgery configurations illustrated with 3D reconstructions of the skull surface based on CT scans. Four burr holes of 15 mm diameter were used in the leftmost example, 7 burr holes of 18 mm diameter in the middle example, and a total elliptic craniectomy of 85 × 65 mm semi-axes in the rightmost example, respectively. In all cases, the skull defects were distributed above the resection cavity and its surrounding borders, as shown in panel B for the leftmost case in panel A. The representation in panel B generally shows the skull and tumor outline in the computational model for the (courtesy of Novocure, Ltd) with the skull surface shown in *purple*, the craniotomy bone flap in *red* (primary surgery) and *dark red* (repeated surgery), the craniotomy line in *pink*, the underlying tumor in *blue*, and the resection cavity in *green*. The transducer array layout is shown in the rightmost illustration in panel B with orange markings on the A/P pair. Panel C shows surface reconstructions of the field distribution before (left) and after (right) SR-surgery for the same patient. Parts of the figure are reproduced from Ref. ^22^. Panel D shows the difference in current density distribution before and after SR-surgery equivalent to the case in panels A (left), B, and C. Results are shown for the skin (left) and CSF (middle) surfaces, respectively, and for the L/R array pair only. It is evident, that a significant amount of current is shunted through the burr holes. The rightmost illustration in panel D shows the equivalent absolute difference in field distribution in the underlying brain. Finally, panel E shows the relationship between the total area of the skull defect for the individual SR-surgery configurations (cm^2^) and the corresponding relative field enhancement (%). Patients treated with TTFields (*n* = 11) are shown in *blue* and patients excluded prior to TTFields (*n* = 4) are shown in *red*.

The objective of the SR-surgery was to maximize the field intensity (ie, dose) in the residual tumor or the region surrounding the resection cavity. The SR-surgery configuration was optimized for each individual (see Figure 2 for examples) by calculating the personalized field distributions before and after different virtual SR-configurations (eg, size, shape, and position). Configurations were explored on a trial-and-error basis. In general, the skull holes were clustered in a relatively small region directly above the resection cavity, to ensure that the TTFields were focused toward the underlying tumor. Due to the complexity of field estimation, with many individual factors influencing the results, it was not feasible to implement a specific dose-escalation regimen (Supplementary Material S2 and Ref. ^22^). Instead, we required the chosen SR-configuration to induce more than 25% enhancement of the average field dose in the tumor to justify any potential risk imposed on the patient due to the SR-surgery. Furthermore, we generally required the total skull defect area to be below 30 cm^2^ as a safety limitation for mechanical brain protection. This area limit was exceeded at the request of 2 patients, as a larger defect induced greater field enhancement in these cases. The SR-surgery design was conducted prior to surgery and implemented with neuronavigation.

Maximum safe resection was performed for all patients, although this was not an inclusion criterion. Surgeries resulting in measurable residual disease on the postoperative MRI (<72 h) according to the RANO criteria were classified as partial resections, whereas surgeries resulting in no residual disease or nonmeasurable residual disease were categorized as gross total resections.

#### TTFields therapy

TTFields therapy was initiated 4 weeks from surgery. Array layouts were planned to maximize the TTFields dose in the tumor.^16^ In a normal clinical setting, the TTFields array layout is planned using the CE-marked and FDA-approved NovoTAL (Novocure) software, which uses individual morphometric measures of the head size and tumor size/position to determine a suitable personalized layout. However, this approach was not appropriate for the present trial, because SR-surgery causes a redistribution of the electric field (Figure 2D), which is not accounted for by NovoTAL. We therefore planned the layouts using more general principles for layout personalization^16,23^ and TTFields dosimetry.^15,24^ Basically, we positioned the arrays such that a row of edge electrodes from one array in each pair overlaid the burr holes and tumor region (Figure 2B). This was done for both pairs so that one array from each pair overlapped the remodeled skull region. The rationale for the approach is that stronger fields are induced at the periphery of the arrays due to the “edge effect,” see Ref. ^16^ and Supplementary Material S3. Therefore, it was not desirable to place holes underneath the central parts of the arrays or far away from the array. The other array in the same pair was placed on the opposite side of the skull so that the line between the paired arrays passed through the target regions of interest. This ensured that both array pairs contributed current flow through the skull holes, inducing high fields in the tumor throughout the entire duty cycle. Given the perpendicular orientation of the 2 array pairs relative to each other, they covered different areas of the underlying tumor and brain region as previously described by Korshoej et al.^23^ The virtual placement of electrodes was performed using the SimNIBS GUI and a custom Matlab script (Mathworks, Inc.).

#### Adjuvant medical treatment

Medical oncological treatment was initiated 2–4 weeks after surgery and included bevacizumab (10 mg/kg every 2 weeks, 3 administrations per cycle) alone or in combination with either lomustine (90 mg/m^2^ every 6 weeks, one administration per cycle) or irinotecan (125 mg/m^2^ every 4 weeks).^25^ Temozolomide rechallenge (200 mg/m^2^) was preferred for patients with MGMT-methylated tumors who had initially completed 6 cycles of adjuvant temozolomide therapy. We did not impose restrictions on supportive care. Corticosteroid administration was reduced to the minimum effective dose.^26,27^

### Endpoint Assessment

The primary endpoint was the severity and frequency of AEs evaluated by the investigators using CTCAEv4.0.^28^

Secondary endpoints were median OS, PFS at 6 months (PFS6), median PFS, OS rate at 12 months (OS12), objective response rate, quality of life (QoL) score (EORTC QLQ-C30 and QLQ–BN20 questionnaires),^29^ cumulative corticosteroid dosage, and KPS decline. Treatment response was evaluated by trained neuroradiologists and neuro-oncologists using the *immunotherapy response assessment in neuro-oncology* (iRANO) criteria,^30^ to allow for the potential delayed response previously demonstrated for TTFields.^27^

### Patient Follow-Up and Monitoring

Clinical examination, including QoL and toxicity assessment, was conducted (1) at baseline, (2) postoperatively during admission, (3) before TTFields and medical oncological therapy, and (4) regularly during adjuvant treatment. MRI, clinical examination, and laboratory tests were conducted every 12 weeks during follow-up, while QoL and toxicity were assessed every 6 weeks.^31^ Additional examinations were conducted upon suspected or validated progression.

### Treatment Discontinuation, Patient Exclusion, and Trial Termination

TTFields therapy was discontinued upon (1) disease recurrence, that is, second overall disease recurrence, (2) grade 3–5 serious AE (SAE) caused by the intervention, or (3) unacceptable AEs regardless of grade. Upon active request from the patient, TTFields therapy beyond progression was allowed on a compassionate use basis or in connection with continued medical treatment. Patients were excluded in the events of (1) death, (2) trial completion, (3) loss to follow-up, (4) withdrawal of consent, or (5) safety prohibiting further participation. The trial was terminated when the final patient was excluded and the necessary data had been acquired. Furthermore, the trial was set to stop in the occurrence of more than 8 SAEs attributed to the intervention.

### Statistical Methods

The trial was exploratory and descriptive so we did not perform sample size calculations nor risk stratification (Supplementary Material S1). AEs were reported as the number and frequencies of patients experiencing a particular AE at least once at any grade. For ongoing AEs with a variable grade over time, for example, the variable intensity of headache, we reported the AE as a single event with the highest grade observed. Time-to-event data (eg, OS and PFS) were calculated from the date of inclusion until the date of the event and censored in the case of patient exclusion. The resulting data were reported using the Kaplan–Meier method with median estimates and 95% confidence intervals (CIs). Binomial data, such as PFS6 and OS12, were reported including 95% CIs using the exact binomial distribution. TTFields compliance rates were calculated as the relative device on-time (%) in the total treatment period. KPS decline was calculated as the absolute difference between the KPS at progression and the KPS at TTFields initiation. The cumulative corticosteroid dose was calculated as a weighted average over the entire inclusion period and expressed in methylprednisolone equivalents. QoL was expressed as the global, functional, and symptom scores according to the EORTC QLQ-C30 and –BN20 guidelines. Electrical field estimates were calculated and reported as described previously in the paper. The relative field enhancement caused by SR-surgery was calculated for each patient, as the increase in predicted field intensity after SR-surgery relative to the field intensity before SR-surgery, that is, (*E*_after_−*E*_before_)/*E*_before_.

## Results

### Patient Flow

**Figure 3.**
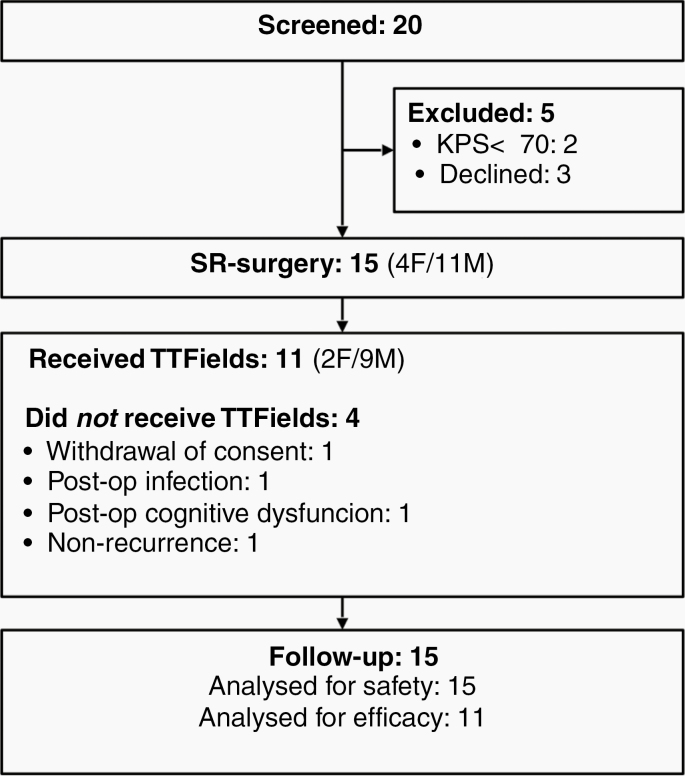
Patient flow diagram.

Twenty patients were screened for participation in the period December 12, 2016 to April 25, 2018. Fifteen were enrolled and followed up until May 31, 2019 (Figure 3). Of those not enrolled, 3 had declined participation, while 2 were excluded due to KPS less than 70. Of the 15 enrolled patients, 4 were excluded prior to TTFields therapy due to radionecrosis/non-recurrence, postoperative infection, neurodeficit (Gerstmann syndrome), and withdrawal of consent, respectively. The remaining 11 patients completed the trial with active TTFields therapy. Baseline data and treatment outline are given in Table 1. The median follow-up period was 14.8 months (range 5.8–25.2 months). All patients were followed up until exclusion and none were lost to follow-up.

**Table 1.** Patient Characteristics and Treatment Outline

Basic Characteristics	Estimate
Age in years, median (range)	57 (39–67)
Male/female (*n*)	9/2
Preoperative KPS, median (range)	90 (70–100)
MGMT methylation (*n*)	4
Tumor location (*n*)	
Frontal	2
Parietal	2
Temporal	5
Parieto-occipital	2
**SR-surgery and TTFields**	**Median (range)**
Skull defect area (cm^2^)	10.5 (7–48)
Field intensity in the tumor (V/m)	173 (111–210)
Relative field enhancement (%)	32 (25–59)
Absolute field enhancement (V/m)	40 (28–69)
TTFields compliance rate (%)	90 (48–98)
TTFields duration (months)	7.6 (2.3–24.0)
**Extent of resection**	***N***
No residual tumor	4
Nonmeasurable residual tumor	5
Measurable residual tumor	2
**Medical treatment**	**Estimate**
Bevacizumab monotherapy (*n*)	8
Bevacizumab/irinotecan (*n*)	1
Temozolomide rechallenge (*n*)	2
Daily methylprednisolone dose in mg, median (range)	14.3 (0–50)

#### Reasons for discontinuation of treatment

TTFields was discontinued for 8 out of the 11 patients. All cases of permanent TTFields discontinuation occurred due to disease progression. AEs did not result in discontinuation for any patient. Four patients continued TTFields therapy beyond progression. Two of these were on a compassionate use basis, while the other 2 also continued active medical treatment with temozolomide and bevacizumab, respectively. Three patients were still on active TTFields at the end of the trial period.

### Baseline Data and Treatment Exposure

We included 11 male patients and 4 female patients. All patients had IDH-*wildtype* tumors. Baseline characteristics of patients treated with TTFields are presented in Table 1 along with the general treatment outline.

#### SR-surgery and tumor resection

For the 15 enrolled participants, the median time from inclusion to SR-surgery and resection was 6 days (range 0–14 days). Figure 2 shows examples of 3 different configurations of SR-surgery of varying extensiveness, including a visualization of the field-enhancing effects induced by the most commonly employed configuration (4 × 15 mm diameter). Field enhancement of more than 25% could be obtained for all patients (Figure 2E). The SR-surgeries were technically feasible, easy to perform, and added less than 15 minutes of additional surgery time. Tumor resection was performed in all cases (see Table 1 for the extent of resection).

Of the 11 patients receiving TTFields, 3 had 4 burr holes of 15 mm diameter, 1 had 5 holes of 15 mm diameter, 4 had 6 holes of 15 mm diameter, 1 had 5 peripheral 15 mm holes and 1 central 25 mm hole, 1 had 7 holes of 15 mm diameter, and 1 had an elliptic craniectomy (semi-axis diameters 85 × 65 mm).

Of the 4 patients excluded before TTFields therapy, 2 had five 15 mm holes, 1 had eight 20 mm holes, and 1 had a total craniectomy (60 × 50 mm). Two of these patients had gross total resection and 2 had nonmeasurable residual tumors.

The relationship between the SR-surgery configuration, for example, the size, arrangement, and number of burr holes, and the induced relative field enhancement was highly complex (Figure 2E). Although a complete review of this relationship was beyond the scope of this study, we generally observed that identical configurations had different efficacy for different patients, for example, 6 × 15 mm burr holes, 10.6 cm^2^, induced field enhancements in the wide range of 25–60%. The field enhancement appeared to plateau around 55–60% when the total area of the skull holes was more than 15–20 cm^2^. There was no linear correlation between the relative field enhancement and the skull hole area (*r*^2^ = 0.55). Similarly, we observed no correlation between the absolute field intensity in the tumor after SR-surgery and the increase in absolute field intensity in the tumor after SR-surgery (*r*^2^ = 0.49 and *r*^2^ = 0.094, respectively). As a part of the scheduled preparation, dosimetry calculations were performed for all patients included in the trial (Figure 2E), that is, also the 4 patients who did not proceed to TTFields therapy. Since all 15 included patients contribute information about the expected field enhancement and the potential correlation with SR size and configuration, we included all patients in the correlation analysis.

#### TTFields therapy and adjuvant medical treatment

Details of the TTFields treatment and adjuvant therapy are given in Table 1. Of all patients receiving TTFields, 8 were treated with bevacizumab monotherapy (median number of cycles = 5, range 2–16). One patient received bevacizumab/irinotecan combination treatment (2 cycles), while 2 patients were treated with temozolomide rechallenge (3 and 4 cycles, respectively). Of the 4 patients not treated with TTFields, 1 received supportive care only, 2 bevacizumab monotherapy (6 and 9 cycles, respectively), and 1 bevacizumab/lomustine combination treatment (7 cycles).

Of those experiencing progression during TTFields (ie, second total recurrence), 2 patients were reoperated, 5 discontinued all treatment, 1 continued bevacizumab with the addition of irinotecan (3 cycles), 1 received temozolomide rechallenge (6 cycles), 1 continued bevacizumab in combination with lomustine (2 cycles), and 1 continued bevacizumab monotherapy (8 cycles). Of the 4 patients not receiving TTFields, 3 discontinued all treatment at progression in the trial (ie, second recurrence) and 1 received 2 cycles of temozolomide before discontinuation.

### Outcomes

#### Adverse events

The observed AEs are presented in Table 2 and Supplementary Table S4 (grading) and Supplementary Table S5 (causality).

**Table 2.** Frequency of Adverse Events

Type of AE	Number of Patients With AEs								
	TTF (*N* = 11)			No TTF (*N* = 4)			Total (*N* = 15)		
	*n*	%	95% CI	*n*	%	95% CI	*n*	%	95% CI
*Neurological*									
Headache	9	81.8	48–98	0			9	60.0	32–84
Speech disturbances	3	27.3	6.0–61	1	25.0	0.6–81	4	26.7	7.8–55
Seizure	5	45.5	17–77	0			5	33.3	12–62
Paresis	1	9.1	0.2–41	3	75.0	19–99	4	26.7	7.8–55
Visual disturbances	2	18.2	2.3–52	0			2	13.3	1.7–41
Neglect	1	9.1	0.2–41	0			1	6.7	0.2–32
Memory disturbances	1	9.1	0.2–41	0			1	6.7	0.2–32
*Regional*									
Skin rash	6	54.5	23–83	0			6	40.0	16–68
Scalp ulceration	2	18.2	2.3–52	0			2	13.3	1.7–41
Surgical wound infection	1	9.1	2.3–52	1	25.0	0.6–81	2	13.3	1.7–41
Surgical wound rupture	2	18.2	6.0–61	1	25.0	0.6–81	3	20.0	4.3–48
Shoulder pain	2	18.2	2.3–52	1	25.0	0.6–81	3	20.0	4.3–48
Axillary abscess	1	9.1	0.2–41	0			1	6.7	0.2–32
*Systemic*									
Fatigue	4	36.4	11–69	2	50.0	6.8–93	6	40.0	16–68
Nausea	3	27.3	6.0–61	3	75.0	19–99	6	40.0	16–68
Fever	3	27.3	6.0–61	2	50.0	6.8–93	5	33.3	12–62
Diarrhea	3	27.3	6.0–61	0			3	20.0	4.3–48
Constipation	2	18.2	2.3–52	0			2	13.3	1.7–41
Abdominal pain	2	18.2	2.3–52	0			2	13.3	1.7–41
Dehydration	1	9.1	0.2–41	0			1	6.7	0.2–32
Deep vein thrombosis	1	9.1	0.2–41	0			1	6.7	0.2–32
Corticosteroid withdrawal syndrome	1	9.1	0.2–41	0			1	6.7	0.2–32
Abnormal ECG	1	9.1	0.2–41	0			1	6.7	0.2–32

The table shows the numbers and frequencies of patients experiencing the observed AEs. A patient is registered as having an AE, if the AE occurred at least once, regardless of severity. All observed AEs were of grades 1–3. Patients are separated into those treated with TTFields (left column) and those not treated with TTFields (middle column). The right column shows results for all patients collectively. Supplementary Tables S4 and S5 list a breakdown of the AE data into grades and causalities, respectively.

In total, we observed 71 AEs of which most were mild to moderate (grade 1 or 2). Considering all patients, 52%, 95% CI: 40–64%, of all AEs were strictly mild with a maximum grade of 1, while 35%, 95% CI: 24–48%, were moderate or below with a maximum grade of 2. We recorded 11 grade 3 SAEs (13%, 95% CI: 7–23%), all unrelated to the intervention, and no grade 4 or 5 SAEs. Most SAEs were neurological (*N* = 5): Three were seizures, which required admission and occurred at progression in patients with prior seizures, 1 experienced a severe headache, while 2 had progressive focal deficits in connection with disease progression. Systemic SAEs were fatigue, deep vein thrombosis, and diarrhea all unrelated to the intervention.

Patients excluded prior to TTFields therapy, that is, within 4 weeks from inclusion, accounted for 19.0%, 95% CI: 11–30%, of AEs. These were mainly neurological deficits attributed to the disease itself or resection surgery, although one patient did experience a grade 3 postoperative infection, which led to exclusion and required surgical revision. None of the surgical AEs could be directly attributed to the SR-surgery per se but rather to the general concept of open surgery, for example, infection, or to tumor resection, for example, postoperative deficits.

Neurological AEs accounted for 37%, 95% CI: 26–49%, of all AEs regardless of grade. The most common neurological AEs were headache (*n* = 9), seizures (*n* = 5), and focal deficits (*n* = 7). Headache was the most frequently observed AE overall, occurring in 60%, 95% CI: 32–84%, of all patients and exclusively in those treated with TTFields. A causal relationship between headache and TTFields was found in 3 cases, while 1 was related to surgery and 5 had unknown causes although unrelated to the intervention. Focal neurological deficits were caused by the disease or resection surgery and all seizures were attributed to disease and progression.

Systemic AEs accounted for 39%, 95% CI: 28–52%, and were generally associated with chemotherapy, GBM, or unknown causes. The most common systemic AEs were fatigue (40% of patients, 95% CI: 16–68%), nausea (40% of patients, 95% CI: 16–68%), and fever (33% of patients, 95% CI: 12–62%). Causality could not be firmly established in 15 of the 29 observed systemic AEs, although these were likely attributed to medical oncological treatment or GBM (eg, fever, nausea, and fatigue). No systemic AE was related to TTFields, 6 were caused by medical oncological treatment (nausea, abdominal pain, and diarrhea), 4 were caused by surgery (postoperative fatigue, nausea, and fever), while 3 AEs had other causes, such as opiate administration or corticosteroid withdrawal.

Regional AEs accounted for 24%, 95% CI: 15–36%, of all cases and mainly comprised grade 1 and 2 skin rash (55% of TTFields-treated patients, 95% CI: 23–83%) and minor scalp ulcerations (18%, 95% CI: 2.3–52%). These manifestations only occurred in patients treated with TTFields and were observed with caution and easily managed with topical corticosteroids and short pauses (1–3 days) in TTFields. One patient had a poor compliance rate of 48% on average due to recurring scalp ulcers. The patient had previously been treated repeatedly with antibiotics for pustulous acne and hidrosadenitis. The ulcers occurred with unchanged frequency and severity and also at distant locations unaffected by TTFields, for example, in the axillary and perineal regions. One patient had a minor postoperative wound rupture with localized infection, which was successfully treated with minor wound revision in local anesthesia and oral antibiotics. This resulted in a 2-week delay of TTFields initiation but did not affect the remaining treatment. One patient in the TTFields group had a minor wound rupture shortly after surgery, which was successfully treated with a single suture. There were no signs of infection and the complications did not affect the medical oncological treatment or TTField.

No surgery inflicted AEs could be associated with the skull remodeling procedure per se, but rather represented common neurosurgical complications, for example, due to resection or complicated wound healing. Within the investigated range of SR-surgery, encompassing minor burr holes and large craniectomies, we did not observe particular toxicity limitations or reach an apparent maximum-tolerated dose neither for the induced field intensity nor for the introduced skull defects.

#### Efficacy outcomes

Survival estimates and efficacy outcomes are presented in Table 3. In general, the results were promising with longer OS (median OS = 15.5 months, 95% CI: 9.4%–NA) and PFS (PFS6 = 36%, 95% CI: 8–64%) estimates compared to historical data although the study was not powered for direct statistical comparison. Kaplan–Meier curves for OS and PFS are shown in Supplementary Figure S6.

**Table 3.** Efficacy Outcome Estimates

Outcome	Estimate
Median OS, months	15.5 months, 95% CI: 9.4–NA
OS at 12 months, %	55%, 95% CI: 25–84
Median PFS, months	4.6 months, 95% CI: 4.1–NA
PFS rate at 6 months, %	36%, 95% CI: 8–64
Objective response rate, %	ORR = 9.1%, 95% CI: 0.2–41.3
Methylprednisolone dose decline, mg	
Total, ie, from inclusion until progression	11.8 ± 19.4 mg
Post-surgery, ie, from inclusion until TTFields initiation	10.4 ± 14.3 mg
TTFields, ie, from TTFields initiation until progression	1.45 ± 12.0 mg

#### Corticosteroid use, KPS, and QoL

The median daily methylprednisolone dose was 14.3 mg (range 0–50 mg) over the course of the trial. The mean total decline in steroid dose during the trial, that is, the presurgical dose at inclusion minus the dose at the time of progression, was considerable (11.8± 19.4 mg) with the most significant reduction occurring after surgery. Accordingly, the presurgical dose at inclusion was reduced by 10.4± 14.3 mg at the time of initiation of TTFields and oncological treatment and further by 1.45 ± 12.0 mg at the time of disease progression (Table 3). Three out of the 11 patients treated TTFields received a high-dose corticosteroid treatment on average (ie, >21.3 mg methylprednisolone daily corresponding to >4 mg of dexamethasone daily), which has previously been shown correlated with poor response to TTFields therapy.^26,27^ Two of these patients experienced progression before 6 months, and 2 died before 1 year of follow-up. The sample size was too low to analyze this correlation.

Eight of the 11 patients treated with TTFields had stable KPS from inclusion until progression (73%, 95% CI: 39–94%). Three experienced a KPS decline of 10–20 points after resection surgery (27%, 95% CI: 6.0–61%). In 2 of these patients, KPS remained stable from surgery until progression (18%, 95% CI: 2.3–52%), while the final patient experienced a KPS recovery of 10 points during TTFields therapy (9.1%, 95% CI: 0.2–41%).

QoL scores were comparable to previous observations,^7^ see Supplementary Table S7. The majority of the patients had high and constant global (median = 141–167) and functional (median = 80–89) QoL scores throughout the trial. Symptom scores were generally low (median = 8.5–11).

## Discussion

In this study, we have evaluated the safety, feasibility, and preliminary efficacy of TTFields in combination with targeted SR-surgery against recurrent GBM. The intervention introduces skull holes in the vicinity of the tumor to facilitate the current flow into the region of interest and thereby enhance the TTFields efficacy.

The intervention was well tolerated and we observed no SAEs directly attributed to the intervention. SAEs were all grade 3 and included fever, fatigue, diarrhea, deep vein thrombosis, as well as seizures occurring at progression in patients with previously known tumor-induced epilepsy.

Overall, the most prevalent AE was headache (60% of patients), whereas minor skin rash of grades 1 and 2 was the most common AE in patients treated with TTFields (55%). These observations correspond well with previously reported rates^10^ and AEs were generally easily managed. The majority of the AEs were systemic (39%, Table 2 and Supplementary Tables S4 and S5) and caused by the disease or the medical oncological treatment.

With regard to efficacy, we observed positive preliminary signals justifying further investigations. The median OS was 15.5 months, 95% CI = 9.4%–NA, which is a considerable improvement compared to the 9 months commonly reported for patients receiving comparable second-line oncological treatment alone.^32,33^ Furthermore, this survival benefit was larger than the expected effects caused by the addition of TTFields alone, although our study was not powered for comparison with historical data. The sole effects of TTFields in the first recurrence of GBM were recently investigated in a post hoc analysis of the randomized EF-14 trial data (NCT00916409).^34^ The study compared the efficacy of TTFields plus physician’s choice chemotherapy with chemotherapy alone after the first recurrence. TTFields prolonged the median OS by approximately 2 months (11.8 months vs 9.2 months, *P* = .049) compared to chemotherapy alone and the authors concluded that the addition of TTFields to medical oncological treatment at first disease recurrence is likely beneficial. This corresponds with observations from the retrospective PRiDe study investigating the real-world application of TTFields therapy in recurrent GBM in the United States.^9^ In the PRiDe cohort, approximately 1/3 of patients received TTFields after the first recurrence and the median OS was 20 months. Collectively, these studies indicate that part of our observations could possibly be explained by TTFields alone and further randomized studies are therefore needed to clarify the potential effects of using SR-surgery to enhance the dose of TTFields. With regard to disease progression, our cohort had a median PFS of 4.6 months and a PFS6 rate of 36%, which is comparable to previous reports of patients treated with comparable second-line medical treatment (median PFS = 3–4.2 months and PFS6 = 16–41%^32,33^).

### Limitations

Despite promising outcomes, our study was subject to important caveats to be considered. Given the small sample size, it was not powered for conclusive estimation, confounder adjustment, or direct comparison with historical data. Furthermore, our cohort was subject to selection bias in the sense that (1) the male/female = 9/2 ratio was higher than expected, (2) the clinical performance was relatively high (median KPS = 90, range 70–100), (3) the rate of gross total resection among TTFields treated patients was high (9/11), and (4) the median TTFields compliance rate was high (90%, range 48–98), of which the latter 3 are known favorable outcome predictors.^9,35^ Therefore, our cohort was expected to perform better than the average population of recurrent GBM patients. Furthermore, 2 nonresponders to TTFields in our cohort were treated with high doses of corticosteroids, which may have potentially reduced the efficacy of TTFields.^26,27^

Also, it should be noted that array placement was based on edge effect considerations and general rules of thumb and not the standard practice NovoTAL approach. Although this approach may have impacted the TTFields efficacy on its own, previous studies indicate that such effects would likely be considerably less compared to those induced by the SR-surgery.^16^

### Future Perspectives

We consider the tested intervention suitable for further investigation, and a randomized controlled phase 2 trial (NCT04223999) is scheduled to begin recruitment in the fall of 2020. The trial includes TTFields in both treatment arms and patients are randomized 1:1 to receive SR-surgery or not. The trial was designed to test the potential for clinical implementation of SR-surgery with TTFields and further to shed additional light on the TTFields dose–response relationship.

Finally, there is a need for further characterization of the effects of different SR configurations, including the number, size, and arrangement of burr holes. We are currently investigating these aspects with a focus on identifying optimal configurations with minimal SR size. Our aim is to establish a feasible SR-surgery approach based on simple rules of thumb and standard operating procedures with priority on clinical utility. These guidelines should also address the aspect of optimal array positioning when using SR-surgery, which differs from the conventional NovoTAL approach, as discussed above.

## Conclusions

SR-surgery offers a rational approach to enhance the TTFields intensity for patients with rGBM and potentially other brain tumors with TTFields sensitivity. The combination of TTFields and SR-surgery is safe and nontoxic and potentially provides individual benefit with the prolonged OS. The trial is the first to prospectively modulate the “dose” of TTFields and investigate its impact on safety and clinical outcome.

## Supplementary Material

vdaa121_suppl_Supplementary-Material-S1

vdaa121_suppl_Supplementary-Material-S2

vdaa121_suppl_Supplementary-Material-S3

vdaa121_suppl_Supplementary-Table-S4

vdaa121_suppl_Supplementary-Table-S5

vdaa121_suppl_Supplementary-Figure-S6

vdaa121_suppl_Supplementary-Table-S7

## Notes

**Authorship statement**. Trial design: A.R.K., S.L., Y.L.R., S.O.S.C., G.v.O., and J.C.H.S. Patient recruitment: A.R.K., G.v.O., and S.O.S.C. Patient treatment, monitoring, and response assessment: A.R.K., S.L., Y.L.R., S.O.S.C., G.v.O., J.C.H.S., K.E.S., and C.R. Data analysis: A.R.K., M.H.J., and N.M. Manuscript drafting: A.R.K., M.H.J., and N.M. Manuscript review: All.

List of unpublished papers: None.
