# Supplementary material for: OptimalTTF-1: Enhancing tumor treating fields therapy with skull remodeling surgery. A clinical phase I trial in adult recurrent glioblastoma
Source: Neurooncol Adv. 2020 Sep 15;2(1):vdaa121. doi: 10.1093/noajnl/vdaa121 (PMC7660275; doi:10.1093/noajnl/vdaa121)
Supplement: vdaa121_suppl_Supplementary-Material-S1 [file vdaa121_suppl_supplementary-material-s1.docx]

**Supplementary Material S1. Additional protocol related aspects.**

*Eligibility assessment and informed consent*

Candidates for participation in the study were identified at bi-weekly institutional multidisciplinary neuro-oncological board meetings. Assessment of recurrence/progression was conducted by board certified and experienced neuro-oncologists, neuroradiologists, neurosurgeons and neurologists in conjunction using the RANO criteria (20). Informed written consent was obtained following eligibility assessment by the investigators from all participants.

*Monitoring and approval*

The trial was monitored by an independent contract research organization and approved by the Central Denmark Region Committee on Health Research Ethics (59954), the Danish Health Authorities (2016023296), and the Danish Data Protection Agency (1-16-02-209-16).

*Technical details of the SR-surgery*

The SR-surgery procedure was outlined in a neuro-navigation plan and performed by a trained neurosurgeon using a Midas Rex surgical drill (Medtronic, Inc). Dural substitutes, hemostatic agents, fibrin glue, and the like were allowed to facilitate hemostasis and dural closure. Burr-hole caps or titanium meshes were not used to avoid diminishing the TTFields enhancement introduced by the SR-surgery. Bone plates were repositioned and fixed to the skull using conventional (small) titanium plates or CranioFix® clamps (B. Braun). Postoperative MRI was performed within 72 hours from surgery to assess the extent of resection.

*Patient monitoring and applied standards*

Patient follow-up was in accordance with the clinical guidelines defined by the Danish Health Authorities and MRI protocols were in accordance with the “consensus recommendations for a standardized brain tumor imaging protocol in clinical trials” (28).

*Toxicity assessment and treatment after exclusion*

Causality and grade of AEs were assessed by the treating physicians and investigators, based on clinical examination and patient record audits. Toxicity evaluation was performed at 30 and 90 days after TTFields discontinuation for all participants and cranioplasty surgery with bone plate replacement was considered, if relevant. Compassionate use of TTFields therapy was allowed beyond progression if requested by the patient and if deemed appropriate by the treating physician.

*Reporting of adverse events*

Adverse events (AEs) were reported in accordance with the “Guidelines on medical devices. Clinical investigations: Serious adverse event reporting, MEDDEV 2.7/3, and Reporting of adverse reactions in clinical trials”.

*Statistical considerations*

The trial was designed to provide a descriptive analysis on a small sample size to identify potential risks of the intervention. Therefore, we did not perform sample size calculations nor risk stratification. Toxicity and adverse events were graded and reported using absolute numbers, proportions and appropriate estimates of central tendencies and statistical dispersion. Statistical analyses were conducted by the Biostatistical Advisory Service at Aarhus University.
