# Supplementary material for: OptimalTTF-1: Enhancing tumor treating fields therapy with skull remodeling surgery. A clinical phase I trial in adult recurrent glioblastoma
Source: Neurooncol Adv. 2020 Sep 15;2(1):vdaa121. doi: 10.1093/noajnl/vdaa121 (PMC7660275; doi:10.1093/noajnl/vdaa121)
Supplement: vdaa121_suppl_Supplementary-Material-S2 [file vdaa121_suppl_supplementary-material-s2.docx]

**Supplementary Material S2. Considerations on field calculations, dose estimation, and escalation planning**

*Dose estimation*

For the purpose of dose estimation and treatment planning, we considered two separate aspects of the intervention, namely 1) the average field intensity induced by TTFields in the tumor and 2) the total area of skull defects introduced with SR-surgery. The field intensity measure correlates with clinical outcome (13) and possibly also with the toxicity of TTFields, independently of the SR-surgery intervention. This is because the SR-surgery only serves to increase the field intensity, while it is not associated with anti-cancer effects by itself. The rationale for quantifying the extent of SR-surgery separately was that the introduced skull defects could potentially be associated with independent surgical complications or risk of traumatic brain injury in the case of head trauma. Furthermore, we also aimed to investigate the level of relative field enhancement that could be attained for particular extents and configurations of SR-surgery.

To evaluate the dose of TTFields therapy, and hence the expected treatment efficacy, we calculated the field intensity distribution individually for all enrolled patients before the SR-surgery was performed. The calculations were performed with and without virtual SR-surgery to assess the potential efficacy of different SR-configurations, see below. For an in depth discussion on the field calculation approach and dose measures on TTFields the reader is kindly referred to Korshoej *et al.*, 2019 (18,25). In general terms, the field calculations were conducted using finite element methods on computational head models constructed from individual patient MRI data. The field distributions were computed for both array pairs separately. We then determined the mean field in the tumor region or peritumoral border zone for each pair and finally calculated the mean intensity of both pairs collectively. The tumor region and resection cavities were outlined manually using a T1 MRI with Gadolinium contrast enhancement. The peritumoral border zone was defined as a 2 cm region of brain tissue around the tumor and resection cavity. The mean field estimate defined the average TTFields dose induced over a treatment duty cycle of 1 second with a 50/50% sequential activation of each pair (duty cycle), equivalent to the clinical treatment setting.

*Considerations on dose escalation*

With regards to dose escalation planning, it is important to consider that the field intensity distribution depends largely on a number of different factors, incl. 1) tumor size, position and morphology, 2) tissue dielectric properties, and 3) morphometric characteristics of the patient's head and brain. Due to variability in these parameters the same SR-surgery procedure could induce different absolute field intensities and different relative field enhancement in two different individuals. For that reason, an equivalent escalation of the size of skull-defects likely leads to incomparable absolute field escalation between individuals. Similarly, equivalent absolute field escalation could require very different extents of SR-surgery between individuals. In short, the absolute field intensity in the tumor does not correlate directly with the extent of SR-surgery, and therefore we found it infeasible to schedule a dose escalation plan for the trial. Instead of aiming for particular dose intervals or escalation schemes, we imposed a minimum requirement on the expected benefit from the surgery (i.e. >25% field enhancement) and a maximum limitation on the skull defect size (30 cm^2^), as explained above, to achieve an appropriate safety/efficacy balance.
