# Supplementary material for: OptimalTTF-1: Enhancing tumor treating fields therapy with skull remodeling surgery. A clinical phase I trial in adult recurrent glioblastoma
Source: Neurooncol Adv. 2020 Sep 15;2(1):vdaa121. doi: 10.1093/noajnl/vdaa121 (PMC7660275; doi:10.1093/noajnl/vdaa121)
Supplement: vdaa121_suppl_Supplementary-Material-S3 [file vdaa121_suppl_supplementary-material-s3.docx]

**Supplementary Material S3. The edge effect of TTFields therapy**

The concept of the edge effect is illustrated in panels A and B of the figure below. This figure shows that stronger fields (A) and current densities (B) are induced near the periphery of the arrays. Results are shown only for a single (left/right) array pair for the sake om simplicity. The configuration is equivalent to the one displayed in Fig. 2 of the manuscript. The influence of the edge effect on the tumor field intensity is illustrated in panels C-E. Panel C shows the average field intensity in a 2 cm diameter tumor with a 1.4 cm diameter central necrotic core. The tumor was virtually introduced at different tumor positions and the field investigated for different array positions. The field distribution was calculated for an orthogonal configuration of two array pairs in the same horizontal plane (B and C) by fifteen-degree stepwise rotations around a central cranio-caudal axis. The tumors were translated at eleven points along an axis between the central transducers of the arrays (left/right) from deep positions (30 mm from the median plane) to superficial positions (50 mm from the median plane) and investigated for all array positions. For all tumors, the maximum average field intensity was achieved when the array pairs were both oriented obliquely at 45 degrees to the sagittal plane (Panel E). The default layout (i.e. anterior/posterior and left/right, panel D) were the least efficient (Korshoej *et al*. 2018 (16)). So, stronger fields are induced when the edge of one array from each pair is placed in close vicinity to the tumor (and the introduced skull defects). The other array in the same pair should be on the opposite side of the head. This approach was also adopted when positioning the arrays in the present trial. The figure is adapted from Korshoej *et al.* 2018 (16).


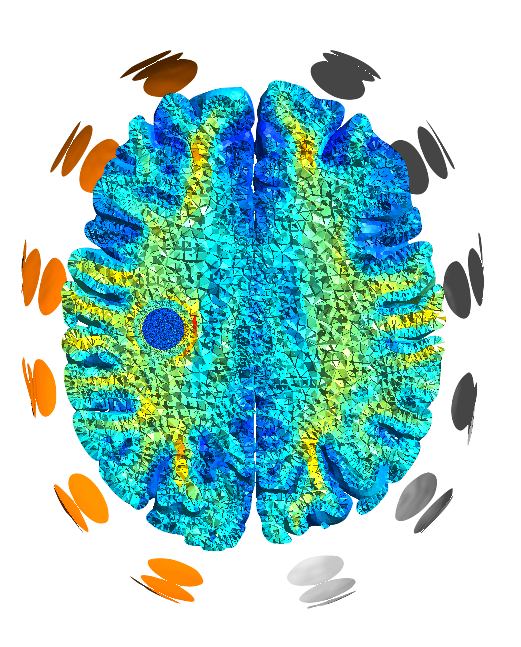

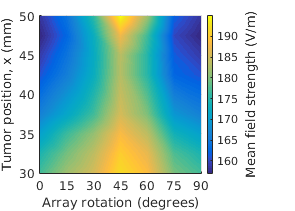

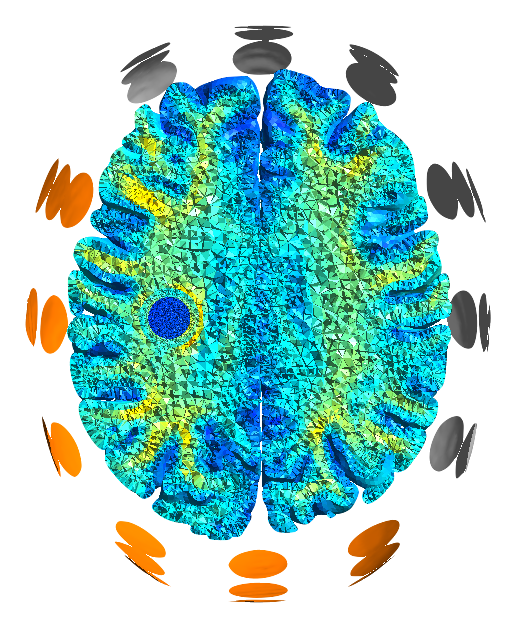


C

Field strength

**two** combined array pairs


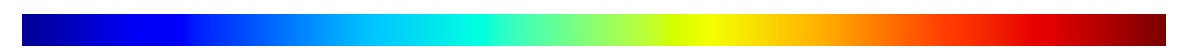


0

450

Field strength (V/m)


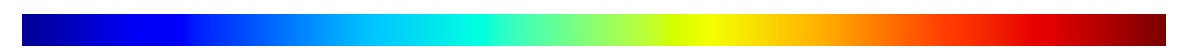


0

450

Field strength (V/m)

Layout 1 (LR/AP)

**Minimum efficacy**

Layout 2 (oblique)

**Maximum** efficacy

D

E


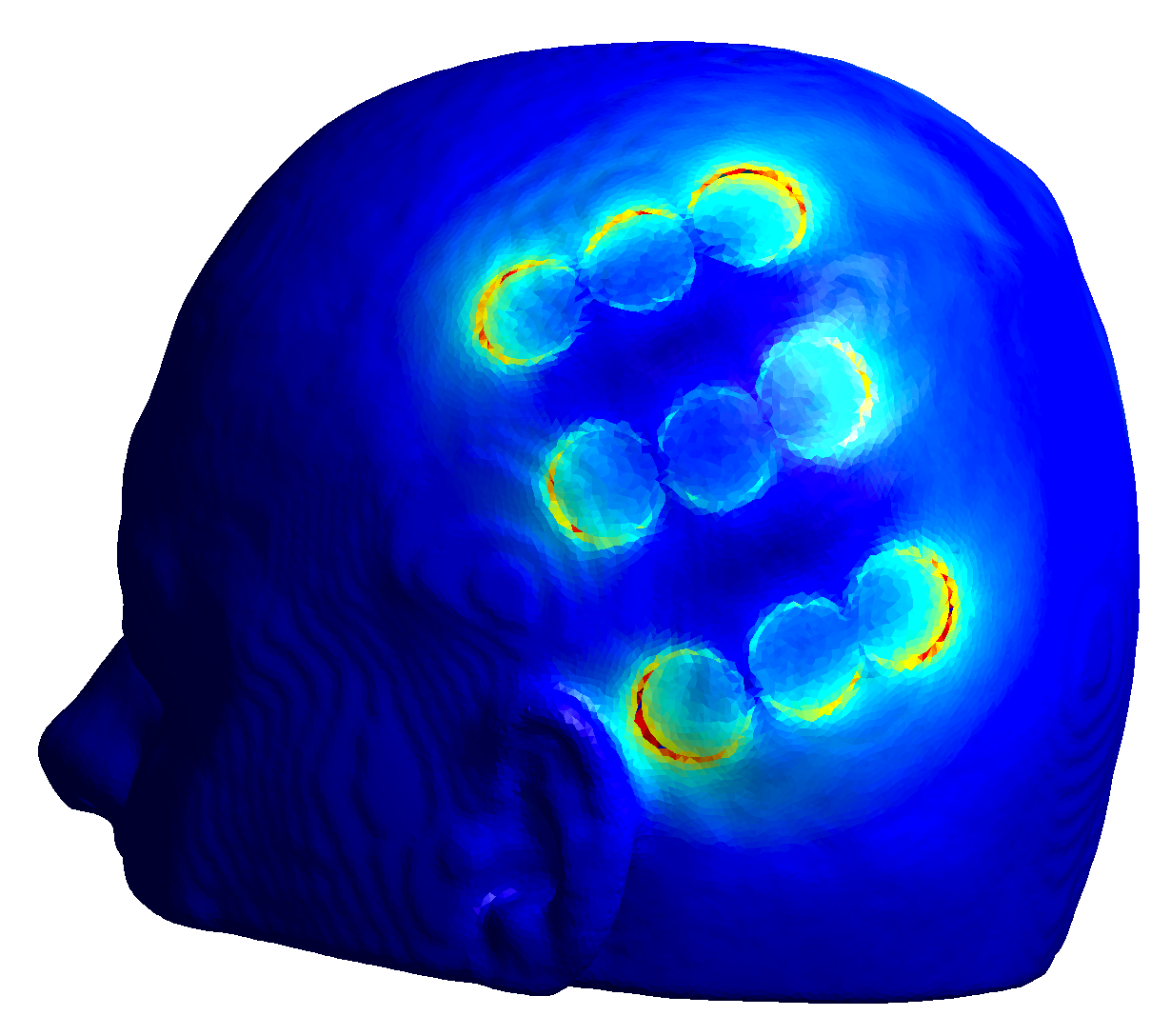

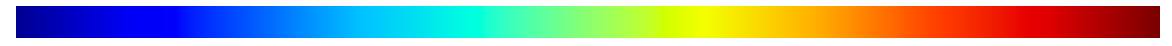


2000

V/m

0


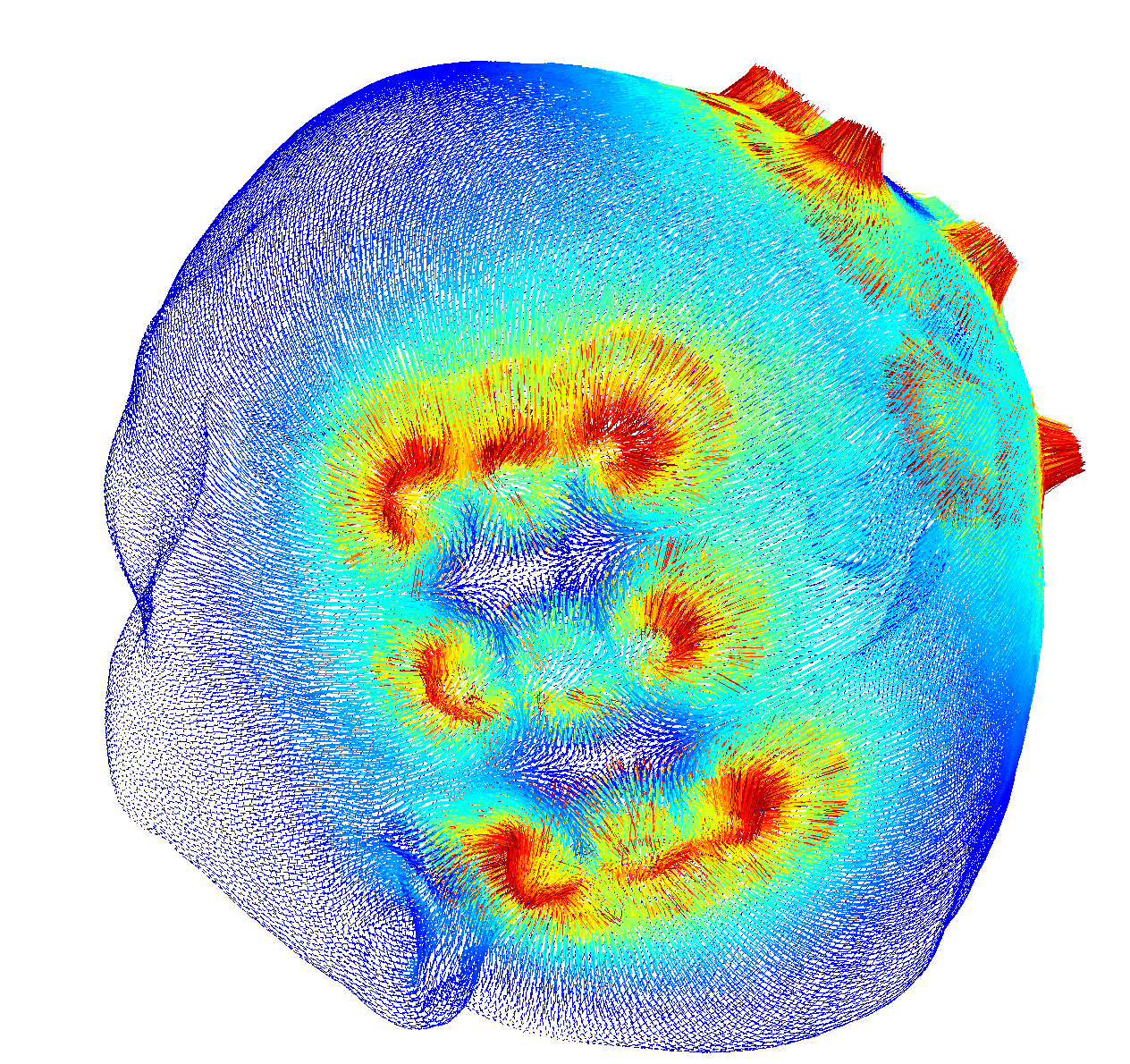

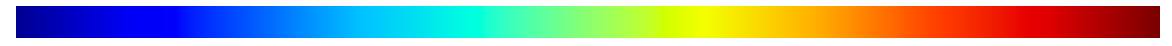


400 A/m^2^

0

A

B
