# Supplementary figures and images for: OptimalTTF-1: Enhancing tumor treating fields therapy with skull remodeling surgery. A clinical phase I trial in adult recurrent glioblastoma
Source: Neurooncol Adv. 2020 Sep 15;2(1):vdaa121. doi: 10.1093/noajnl/vdaa121 (PMC7660275; doi:10.1093/noajnl/vdaa121)

**Supplementary Table S4. Grading of adverse events
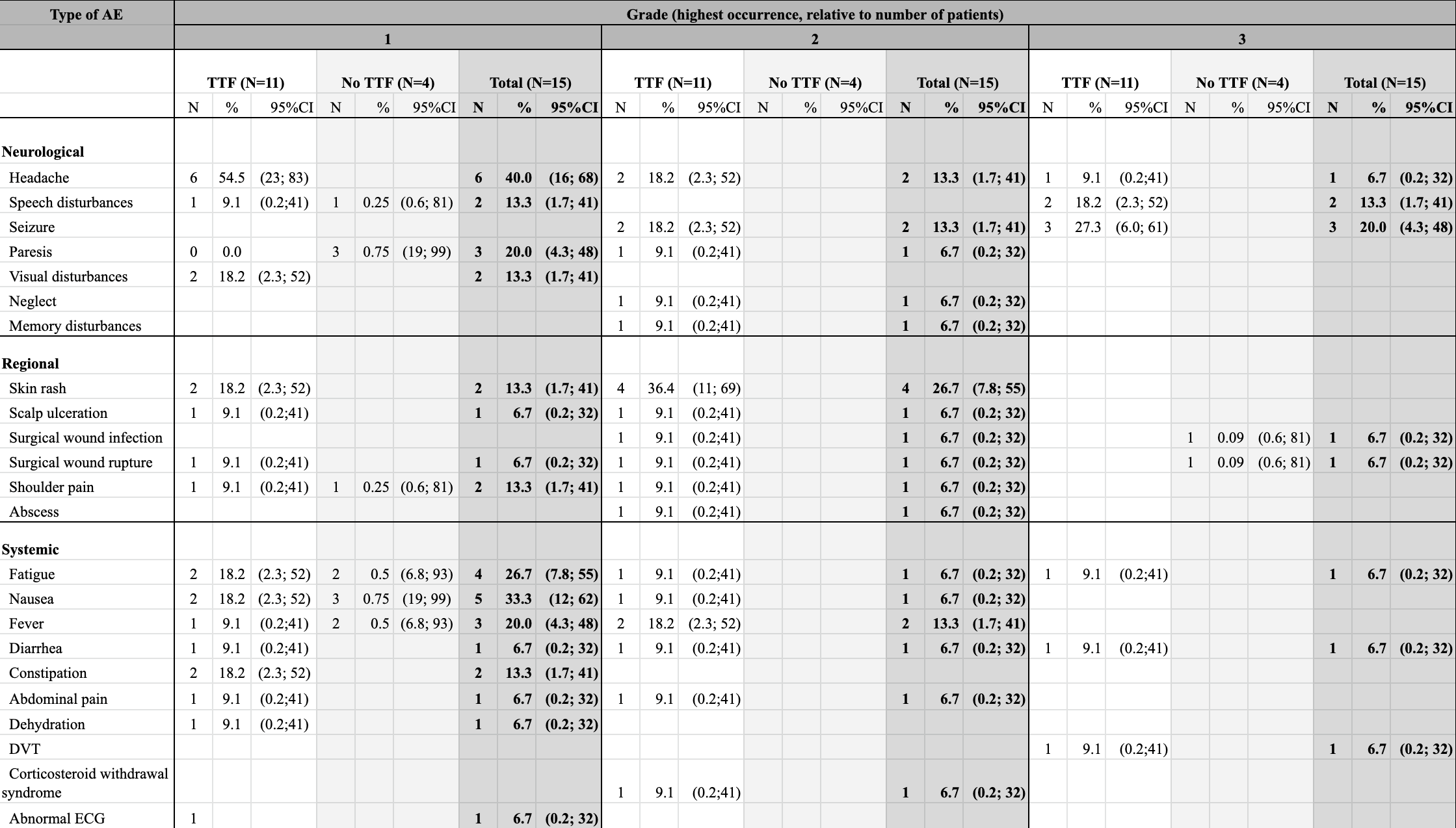
**

Supplement: vdaa121_suppl_Supplementary-Table-S4 [file vdaa121_suppl_supplementary-table-s4.docx]

**Supplementary Table S5. Causality of adverse events**

**
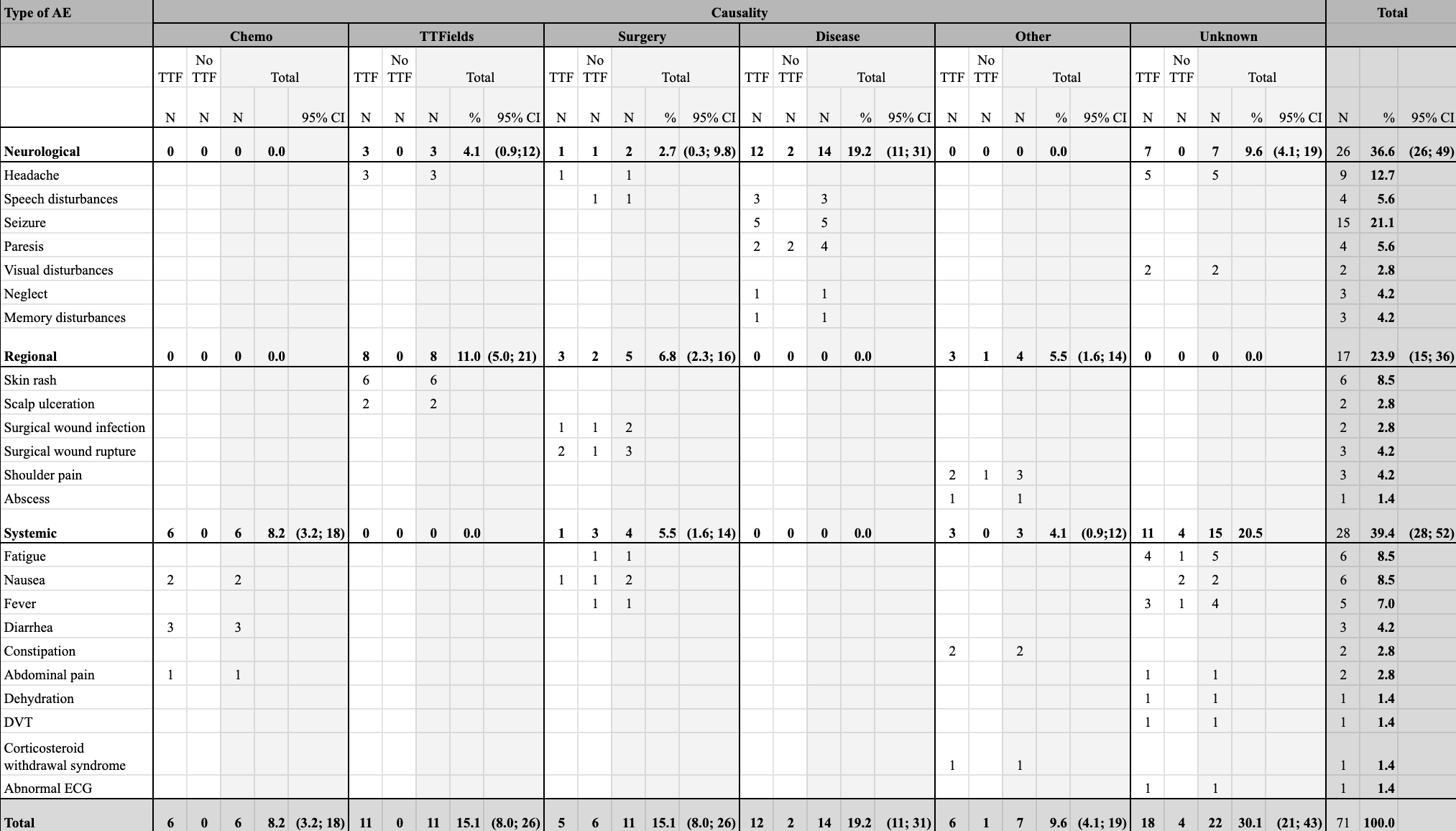
**

Supplement: vdaa121_suppl_Supplementary-Table-S5 [file vdaa121_suppl_supplementary-table-s5.docx]
