# Supplementary material for: OptimalTTF-1: Enhancing tumor treating fields therapy with skull remodeling surgery. A clinical phase I trial in adult recurrent glioblastoma
Source: Neurooncol Adv. 2020 Sep 15;2(1):vdaa121. doi: 10.1093/noajnl/vdaa121 (PMC7660275; doi:10.1093/noajnl/vdaa121)

**Supplementary Figure S6. Kaplan-Meier curves showing progression-free survival and overall survival for all patients in the trial.** The solid lines represent the Kaplan-Meier curves, the stippled lines represent the 95% confidence limits and censored observations are shown with tick-marks.
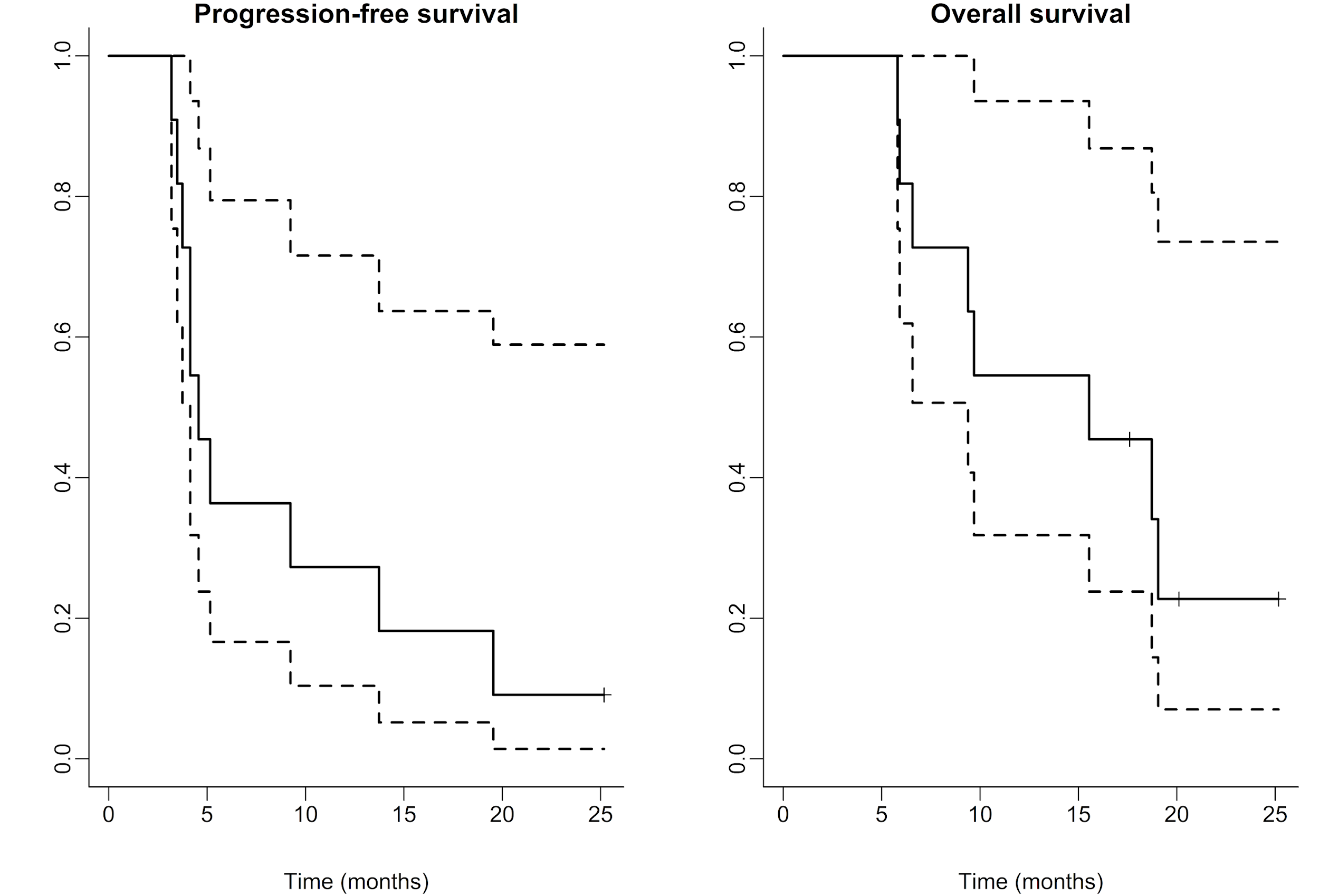

Supplement: vdaa121_suppl_Supplementary-Figure-S6 [file vdaa121_suppl_supplementary-figure-s6.docx]
