# Supplementary material for: OptimalTTF-1: Enhancing tumor treating fields therapy with skull remodeling surgery. A clinical phase I trial in adult recurrent glioblastoma
Source: Neurooncol Adv. 2020 Sep 15;2(1):vdaa121. doi: 10.1093/noajnl/vdaa121 (PMC7660275; doi:10.1093/noajnl/vdaa121)
Supplement: vdaa121_suppl_Supplementary-Table-S7 [file vdaa121_suppl_supplementary-table-s7.docx]

| Supplementary Table S7. Quality of life based on QLQ-C30 and -BN20 questionnaires. | | | |
| --- | --- | --- | --- |
| Time | **Global score** | **Functional score** | **Symptom score** |
| Baseline, median (range) | 141.1 (50-200) | 88.8 (62.2-97.2) | 11.1 (4.0-25.3) |
| TTF start, median (range) | 166.7 (50-200) | 85.5 (62.2-95.5) | 8.5 (6.1-14.1) |
| Progression, median (range) | 166.7 (50-200) | 80 (48.4 - 91.1) | 10.1 (0-42.4) |
